# Ca^2+^ Transportome and the Interorganelle Communication in Hepatocellular Carcinoma

**DOI:** 10.3390/cells11050815

**Published:** 2022-02-26

**Authors:** Hong-Toan Lai, Reynand Jay Canoy, Michelangelo Campanella, Yegor Vassetzky, Catherine Brenner

**Affiliations:** 1 CNRS, Institut Gustave Roussy, Aspects Métaboliques et Systémiques de l’Oncogénèse pour de Nouvelles Approches Thérapeutiques, Université Paris-Saclay, 94805 Villejuif, France; hongtoan.lai@gmail.com (H.-T.L.); rccanoy@up.edu.ph (R.J.C.); m.campanella@ucl.ac.uk (M.C.); yegor.vassetzky@igr.fr (Y.V.); 2 Institute of Human Genetics, National Institutes of Health, University of the Philippines, Manila 1000, Philippines; 3 Department of Comparative Biomedical Sciences, The Royal Veterinary College, University of London, London NW1 0TU, UK; 4 Consortium for Mitochondrial Research, University College London, London WC1 0TU, UK; 5 Department of Biology, University of Rome Tor Vergata, 00133 Rome, Italy

**Keywords:** hepatocellular carcinoma, Ca^2+^ transportome, metabolic reprogramming, interorganelle communication

## Abstract

Hepatocellular carcinoma (HCC) is a type of liver cancer with a poor prognosis for survival given the complications it bears on the patient. Though damages to the liver are acknowledged prodromic factors, the precise molecular aetiology remains ill-defined. However, many genes coding for proteins involved in calcium (Ca^2+^) homeostasis emerge as either mutated or deregulated. Ca^2+^ is a versatile signalling messenger that regulates functions that prime and drive oncogenesis, favouring metabolic reprogramming and gene expression. Ca^2+^ is present in cell compartments, between which it is trafficked through a network of transporters and exchangers, known as the Ca^2+^ transportome. The latter regulates and controls Ca^2+^ dynamics and tonicity. In HCC, the deregulation of the Ca^2+^ transportome contributes to tumorigenesis, the formation of metastasizing cells, and evasion of cell death. In this review, we reflect on these aspects by summarizing the current knowledge of the Ca^2+^ transportome and overviewing its composition in the plasma membrane, endoplasmic reticulum, and the mitochondria.

## 1. Introduction

Liver cancer poses a major challenge to the national healthcare system of many countries, particularly in east Asian countries [1,2]. Among the primary liver cancer types, hepatocellular carcinoma (HCC)—an extremely malignant tumour—is one of the most predominant (approximatively 80%) and accounts for 1% of all deaths across the world [3]. HCC is associated with the four most common predisposing factors, which include the hepatitis B virus, hepatitis C virus (HCV), non-alcoholic fatty liver disease (NAFLD), and alcoholic liver disease (ALD). As the third most common cause of death from cancer in the Asia–Pacific region, 72% of HCC cases can be found in Vietnam, China, North Korea, and South Korea [4]. In these populations, the male-to-female ratio reaches up to 3:1, hence the HCC rate in men is three times higher than in women [5,6]. This epidemiological information suggests that the different genetic backgrounds can explain the heterogeneity between Asia–Pacific regions and sub-Saharan Africa with the rest of the world [7]. According to the Barcelona Clinic Liver Cancer (BCLC) staging system, HCC is diagnosed mostly at the intermediate and advanced stages (BCLC B, BCLC C, and BCLC D), with high mortality rates [8]. Only early-stage (BCLC 0 and BCLC A) patients are eligible for conventional treatments, such as local ablation, surgical resection, or liver transplant [9,10]. Another crucial clinical obstacle is that HCC is particularly resistant to chemotherapy, due to multidrug resistance (MDR) mechanisms induced by conventional anticancer drugs. Reduced drug uptake observed in HCC is due to the upregulation of drug efflux pumps, such as multidrug resistance-associated protein 2 (MRP2) and multidrug resistance 1 (MDR1) [11,12]. Ca^2+^ is indicated to play part in the formation and progression of HCC, being implicated in the regulation of multiple hepatic functions, including lipid and carbohydrate metabolism, as well as bile secretion.

Ca^2+^ is a versatile signalling messenger that regulates vital as well as lethal cellular functions, spanning gene expression, metabolism, apoptosis, muscle excitation-contraction, and neurotransmission [13]. The influx and efflux of Ca^2+^, both at the cellular and intraorganellar level, depend on many ion transporters and exchangers that are collectively referred to as the Ca^2+^ transportome. The Ca^2+^ transportome is finely regulated [14], and its aberration disrupts Ca^2+^ homeostasis, thus contributing to tumour proliferation [15]. Finally, Ca^2+^ is well known as a mediator of interorganelle communications and most important metabolic processes, including the production of reactive oxygen species (ROS), ATP, oncoproteins, and oncometabolites [16].

The deregulation of the Ca^2+^ transportome has been actively studied in recent years, showing its contribution to tumor development in several cancers. Thus, in oncogenesis, remodelling Ca^2+^ transportome activity is required to shift the balance between cell life and death towards the development of many cancer types, including HCC [17]. In most non-excitable cancer cells, such as prostate, breast cancers, stromal interaction molecule (STIM) and/or the Orai protein-mediated store-operated Ca^2+^ entry (SOCE) mechanism, are generally downregulated to avoid cytosolic Ca^2+^ overload, thus evading cell death and promoting tumor proliferation [18,19]. Moreover, the SOCE mechanism is also deregulated in glioblastoma, melanoma, and renal cell carcinoma, resulting in tumor invasion, migration, and metastasis [20,21,22]. Plasma membrane-permeable transient receptor potential (TRP) channels, such as TRP Vanilloid subfamily member 6 (TRPV6) and TRP cation channel subfamily C member 6 (TRPC6), are shown to contribute to prostate cancer proliferation [23,24]; alternatively, TRP cation channel subfamily C member 1 (TRPC1) plays a role in promoting the non-small-cell lung carcinoma cell cycle [25]. The inositol 1,4,5-triphosphate (InsP_3_) receptor (IP_3_R)-mediated endoplasmic reticulum–mitochondria crosstalk is also involved in the apoptosis resistance of glioblastoma, thus revealing the role of ER and mitochondrial calcium in tumorigenesis [26]. At the mitochondrial level, the mitochondrial Ca^2+^ uniporter (MCU) can regulate tumor progression in breast cancer and colorectal cancer growth [27,28], whereas the mitochondrial Ca^2+^ uptake 1 (MICU1) protein drives chemoresistance in ovarian cancer [29].

Here, we provide an overview of the Ca^2+^ transportome composition and, secondly, the impact of its deregulation on HCC, via interorganelle communication, based on the latest studies. We will discuss recent evidence revealing that some members of the Ca^2+^ transportome (i.e., MCU, MCU regulator 1 (MCRU1), TRPC6, and STIM1) notably impact intracellular Ca^2+^ level, mitochondrial ROS (mtROS) production, and transcriptomic profiles, and how these deregulations specifically promote HCC progression.

## 2. Ca^2+^ Transportome Composition and Function

### 2.1. Plasma Membrane Ca^2+^ Transportome

The plasma membrane (PM) defines the boundary separating the intracellular environment and the extracellular space. Following the fluid mosaic model [30], the PM harbours sites with different biophysical properties, contributing to the dynamic function of transporters and exchangers that mediate the communication between the internal and the external [31]. Since cytosolic Ca^2+^ signalling controls crucial cellular functions, basal Ca^2+^ concentration must be tightly regulated and maintained at very low levels [32]. Thus, the continuous influx and efflux of Ca^2+^ between the extracellular environment and the cytosol are mediated by the PM Ca^2+^-permeable ion channels and energy-dependent channels described below.

#### 2.1.1. Ca^2+^-Permeable Ion Channels of the Plasma Membrane

**Figure 1:**
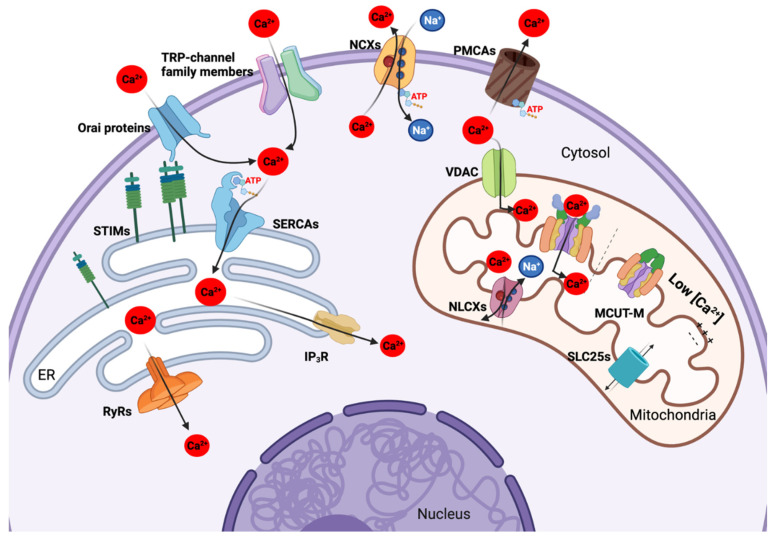
Hepatocellular carcinoma Ca^2+^ transportome located in the plasma membrane (PM), endoplasmic reticulum (ER), and mitochondria. Ca^2+^ ions are transported from extracellular environment into cytosol mainly via two PM Ca^2+^-permeable ion channels, called the TRP channel and SOC channel represented by Orai proteins. On the other hand, the extrusion of cytosolic Ca^2+^ is executed by two PM energy-dependent Ca^2+^ channels: NCXs (antiporters transporting Na^+^ ions against Ca^2+^ ions) and PMCA. PM can also interact with ER via STIM/Orai complexes that induce the Ca^2+^ influx inside the cytosol. Ca^2+^ accumulation inside the ER is mediated by an energy-dependent pump called SERCA. RyRs and IP_3_R are responsible for Ca^2+^ release from ER storage into cytosol, thus regulating many Ca^2+^-dependent signalling pathways. In mitochondria, Ca^2+^ uptake is mainly conducted through VDAC (mitochondrial outer membrane) and MCUT-M (mitochondrial inner membrane). In normal physiological conditions, MCUT-M is only open when the Ca^2+^ concentration in intermembrane space is high. The extrusion of Ca^2+^ out of mitochondria is mediated by NCLXs and SLC25s members. Several SLC25s members can also be regulated by mitochondrial Ca^2+^. Figure created with BioRender.com.

PM Ca^2+^-permeable ion channels are passive transporters, since the Ca^2+^ flux is electrochemical gradient-dependent and does not consume energy. There are seven subclasses of PM Ca^2+^ permeable channels in both excitable and non-excitable human cells, including: (1) voltage-gated Ca^2+^ channels (VGCC or Ca_v_), (2) ligand-gated Ca^2+^ channels (LGC), (3) store-operated channels (SOC), (4) transient receptor potential (TRP) channels, (5) second messenger-operated channels (SMOC), (6) acid-sensing ion channels (ASIC), and (7) mechano-gated channels [17]. In normal hepatocytes PM, only the TRP- and SOC- channel families are expressed to mediate Ca^2+^ transport (Figure 1).

The TRP channel family is a large family of conserved cation-permeable channels that sense exogenous and endogenous stimuli (pH, osmolarity, temperature, etc.) to mediate Ca^2+^ entry [33]. Many TRP channel subfamilies, such as TRPC, TRP melastatin (TRPM), and TRPV, have been identified as valuable biomarkers for diagnosis, as well as potential targets for pharmaceutical treatment, over the past few decades [34]. In HCC, the most studied TRP member is TRPC6 (106 kDa), which is responsible for migration, invasion, and drug resistance, and is further discussed below [35,36]. SOCs are represented by the family of Orai proteins. Mouse and human genomes contain three paralogs: *ORAI1*, *ORAI2,* and *ORAI3*. Orai1 was the first member to be discovered (in 2006 by RNA interference), and is also the most well-known channel to contribute to the SOCE mechanism [37,38,39]. Orai1 is a 33 kDa PM surface protein with four transmembrane domains, located between the N-terminal and C-terminal domains [40]. The characteristic that makes Orai proteins crucial is their very high sensitivity for Ca^2+^. The SOCE mechanism requires the physical interaction between the C-terminal of the Orai proteins with the ER STIM1 (described below), thus inducing the conformational change of the Orai proteins and allowing Ca^2+^ influx when the ER Ca^2+^ level is depleted [41,42]. In normal rat and mouse hepatocytes, Ca^2+^ influx is mainly mediated by the SOCE mechanism via SOCs, represented by Orai proteins. In HCC cell lines (e.g., Huh-7 and HepG2), TRP channels can also interact with Orai1 and STIM1, thus contributing to SOCE regulation and promoting tumour proliferation [43]. This classical model of STIM1 and Orai1 has been extensively implicated in tumorigenesis of many cancers, including hepatoma, breast, colorectal, prostate, etc., Orai proteins and STIM proteins represent attractive therapeutic targets [44].

To date, there has been no observation of functional VGCCs in normal hepatocytes, since VGCCs are present mostly in excitable cells (i.e., neuron, muscle, or neuron-like cell types) [45]. Surprisingly, VGCCs seem to be present in liver cancer stem cells and HCC [46,47]. Moreover, the α2δ1 subunit of many VGCCs has recently been identified as a novel biomarker for HCC diagnosis [48], and is thought to maintain the stem cell-like characteristics of HCC [49].

#### 2.1.2. Energy-Dependent Ca^2+^ Channels and Ca^2+^ Extrusion Systems of the Plasma Membrane

Two systems of Ca^2+^ extrusion include the high-affinity, low-capacity Ca^2+^-ATPase, also known as the PM Ca^2+^ pump (PMCA), and the low-affinity, high-capacity Na^+^/Ca^2+^ exchanger (NCX) [50] (Figure 1), discovered in the 1960s as critical regulators of cytosolic Ca^2+^ levels [51,52,53].

The PMCA carries out the ATP-dependent export of Ca^2+^. This pump is predicted to contain 10 transmembrane domains with two loops that contain a phospholipid-binding domain and an ATP-binding site for its activation [54]. The calmodulin-binding domain, adjacent to the C-terminus, plays a role in PMCA autoinhibition at low cytosolic Ca^2+^ concentrations [55]. The PMCA is encoded by four different genes; *PMCA1* and *PMCA4* are housekeeping genes, *PMCA2* and *PMCA3* are tissue-specific [56]. Alteration of PMCA mRNA and protein levels is observed in many types of cancers, including melanoma, gastric, and oral cancers [57,58,59]. Although the PMCA1 (134 kDa) protein level is downregulated in skin, lung, and oral cancer, it is upregulated in breast cancer and murine hepatoma cells, leading to questions as to whether it participates in cancer proliferation [60,61].

NCX mediates Ca^2+^ extrusion by entering three Na^+^ ions, and extruding one Ca^2+^ ion, against a gradient [62]. Its structure is predicted to have nine transmembrane domains. The first five transmembrane domains, located in the N-terminus, are separated from the remaining four transmembrane domains, in the C-terminus, by a cytosolic loop that contains two Ca^2+^ binding domains (CBD1 and CBD2) that are important for its regulation [50,63]. The human NCX family includes three members encoded by three distinct genes: *NCX1*, *NCX2*, and *NCX3* [64]. According to several studies, under specific conditions, NCX can also function in a reverse mode, in which NCX mediates Ca^2+^ influx and Na^+^ efflux [65]. Among these three members, NCX1 (109 kDa) is the most characterized exchanger. As mentioned above, any change in the cytosolic Ca^2+^ level can affect the growth of cancer cells as efficiently as it affects normal cell growth. Unfortunately, the contribution of NCX members to HCC remains under-studied [66].

### 2.2. The Ca^2+^ Transportome of the Endoplasmic Reticulum

The endoplasmic reticulum (ER) was described by K.R. Porter in 1945 as a cytoplasmic “lace-like reticulum” structure connected to the nuclear envelope [67]. Different ER subregions include rough (around the nucleus for the synthesis of secreted proteins), smooth (metabolism and Ca^2+^ signalling), and transitional ER (close to the Golgi apparatus for post-translational modification) [68]. The ER is essential for numerous physiological functions (synthesis of secreted proteins, metabolism, cell death, etc.) [69]. It is the main compartment in which the majority of intracellular Ca^2+^ is stored, its Ca^2+^ content depending on the cooperation of various channels belonging to the ER Ca^2+^ transportome, such as the ryanodine receptor (RyR) and the IP_3_R as Ca^2+^ release channels, and the SERCA family for ER Ca^2+^ accumulation (Figure 1).

The ER forms membrane contact sites (MCS) with the mitochondria, PM, and endosomes to mediate Ca^2+^ fluxes [70,71,72,73]. Thus, the ER lumen is able to contain high concentrations of Ca^2+^ (~100 µM), whereas the cytosolic Ca^2+^ concentration is in the 50–100 nM concentration range [74]. The trafficking of Ca^2+^ between the ER and the other compartments is strictly regulated, as leakage of ER luminal Ca^2+^ can cause cell death by mediating ER stress and activating unfolded protein responses (UPR) [75]. The release of ER Ca^2+^ into the cytosol is crucial to mediate Ca^2+^-dependent signalling pathways; however, its over-release can induce ER Ca^2+^ depletion [72,73]. In order to avoid ER Ca^2+^ depletion, the SOCE mechanism is activated to enhance the influx of cytosolic Ca^2+^ through the PM via STIM proteins and PM Orai proteins [76]. Thus, a better understanding of the ER Ca^2+^ transport is crucial to visualize the impact of the ER on the regulation of cytosolic Ca^2+^ levels and Ca^2+^ signalling pathways in HCC.

#### 2.2.1. Ca^2+^ Permeable Efflux Transporters in the ER

ER Ca^2+^-permeable channels are categorized into two types according to two mechanisms: (1) Ca^2+^-induced Ca^2+^ release (CICR), and (2) agonist-induced G protein-coupled receptor (GPCR)-dependent release [17] (Figure 1).

The ryanodine receptor (RyR) family is known to comprise the major Ca^2+^ channels that mediate CICR process [77]. Three isoforms, with three distinct functional properties, of RyR are encoded by homologous genes and form the following structural homotetramers: RyR1 (565 kDa) (skeletal muscle cells), RyR2 (565 kDa) (cardiac muscle cells), and RyR3 (552 kDa) (ubiquitous but unclear physiological role) [78]. The main ligand of the RyR channels is ryanodine, and cyclic ADP-ribose (cADPR) for RyR2 and RyR3 only. It is believed that RyR channels participate in regulating the Ca^2+^ signalling pathway in myocytes; however, their roles in hepatocytes are less known [79]. Nicola et al. demonstrated that, in normal hepatocytes, ryanodine and cADPR (two known agonists of RyRs) can facilitate ER-mediated Ca^2+^ release into the cytosol. However, these authors only isolated the RyR1 isoform, without the N-terminal mRNA sequences, which retains its normal function as a Ca^2+^ transporter [80]. Moreover, recent HCC genomic analysis revealed the high mutation rates found in Ca^2+^ transporters, including RyR1 and RyR2 genes [81].

Adjacent to CICR, agonist-induced GPCR-dependent Ca^2+^ release also regulates intracellular Ca^2+^ homeostasis in hepatocytes. IP_3_Rs are known to be involved in this process. There are three isoforms of IP_3_R, named IP3R1 (314 kDa), IP_3_R2 (308 kDa), and IP_3_R3 (304 kDa), encoded by homologous genes. To activate IP_3_Rs, phospholipase C (PLC) breaks down phospholipid phosphatidylinositol 4,5-biphosphate (PIP _gm_) to generate diacylglycerol and InsP3 (a second messenger that binds to IP_3_Rs and causes conformational change, and thus ER Ca^2+^ release) [82]. Among the three isoforms, IP_3_R3 was found to be absent or poorly expressed in normal hepatocytes, but was overexpressed in HCC patients and HCC cell line models. This overexpression enhances the Ca^2+^ signalling in HCC, and prevents apoptosis [83]. Moreover, the IP_3_R mediates the Ca^2+^ transport from the ER into mitochondria via the chaperone glucose-regulated protein 75 (Grp75) and voltage-dependent anion channel (VDAC), creating the so-called ER–mitochondrial contact sites [84].

#### 2.2.2. Energy-Dependent ER Ca^2+^ Influx Transporters

Sarco/ER Ca^2+^-ATPases (SERCAs) are ER transmembrane proteins, encoded by *ATP2A1*, *ATP2A2*, and *ATP2A3* genes, represented, respectively, by three isoforms SERCA1 (110 kDa), SERCA2 (114 kDa), and SERCA3 (109 kDa) [85]. SERCAs contain four major domains, which are responsible for the accumulation of Ca^2+^ into the ER by transporting Ca^2+^ ions against the concentration gradient, as described for the first time by Toyoshima and colleagues [85,86]. This physiological function maintains the Ca^2+^ concentration balance between the ER and cytosol, as shown by the use of SERCA inhibitors, such as thapsigargin [85]. In fact, the alternative splicing of *ATP2A1*, *ATP2A2*, and *ATP2A3* genes resulted in over 70 SERCA-derived isoforms, which contribute differently to various types of cancer cells [87]. Additional to Ca^2+^ homeostasis, SERCAs also regulate cell survival and the ER stress signalling pathway [88,89]. For example, the overexpression of SERCA2 correlates with a higher tumour grade in colorectal cancer, suggesting the presence of an apoptosis resistance mechanism by avoiding cytosolic Ca^2+^ overload [90]. On the contrary, Xia and collaborators gave recently shown that the downregulation of SERCA2 in HCC avoids anoikis and promotes its metastasis [91].

#### 2.2.3. Stromal Interaction Molecule 1 (STIM1)-Mediated Store-Operated Ca^2+^ Entry (SOCE) Mechanism

The SOCE mechanism is present only in non-excitable cells. The channels that participate in this mechanism only open when the ER Ca^2+^ concentration is low, in order to refill the Ca^2+^ stock and promote the Ca^2+^ signalling pathways. SOCE mainly involves two actors: the STIM and the Orai channel [17]. STIM proteins were separately identified in *Drosophila melanogaster* and in humans in 2005, with two isoforms reported, STIM1 and STIM2 [44]. It has been demonstrated that STIM1 is involved in PM Ca^2+^ influx when the ER Ca^2+^ concentration is decreased [92]. When the basal ER Ca^2+^ level is high, STIM1 is inactive. In contrast, when the ER Ca^2+^ level is low, STIM1 is translocated within the ER to PM proximal sites to stimulate PM Ca^2+^ entry [93]. The target Ca^2+^ channel of STIM1 activation is named Orai1; STIM1 and Orai1 physically interact with each other and promote the SOCE-mediated PM Ca^2+^ influx [38]. The structure of STIM1 contains EF-hands that can sense the Ca^2+^ level changes in the ER, thus keeping STIM1 in an inactive state when the ER Ca^2+^ level is high, and in an active state when the ER Ca^2+^ level is low. Recently, many groups have investigated the deregulation of STIMs and Orai proteins in a wide range of cancers. Interestingly, STIM1 is overexpressed in several types of tumours, and is therefore acknowledged to be a promoter of tumour migration and invasion, thus standing as an attractive target for controlling tumorigenesis [92].

### 2.3. Mitochondrial Ca^2+^ Transportome

The mitochondria were described for the first time in the 1840s; later, in 1912, Otto Heinrich and Warburg linked this organelle to cellular respiration [94]. The concept of the “powerhouse of the cell” is now widely accepted to be responsible for producing ATP and modulating many biosynthetic intermediates. Mitochondria form a dynamic, interconnected network with other cellular organelles to maintain cellular homeostasis. Furthermore, mitochondria, which are in physical contact with the ER, lysosomes, and the nucleus [95,96,97], play an important role in Ca^2+^ regulation at a spatio–temporal level. Alterations in the mitochondrial Ca^2+^ concentration directly impact OXPHOS function, ATP production, as well as cell death execution. Thus, cytosolic Ca^2+^ propagates into the mitochondria and leads to an enhanced respiration rate, H^+^ extrusion, ATP synthesis, ultimately changing the whole dynamic of mitochondrial metabolism and energy production. Cancer cells have the tendency to remodel mitochondrial Ca^2+^ homeostasis to facilitate tumour development.

Mitochondrial Ca^2+^ uptake is conducted via VDAC in the outer mitochondrial membrane (OMM), and via the MCU in the inner mitochondrial membrane (IMM) (Figure 1). Interestingly, VDACs can transport many other molecules and metabolites (MM < 5 kDa) and are functionally regulated by Ca^2+^. The MCSs associated with the ER are also an important feature for optimizing Ca^2+^ uptake inside the mitochondria (see above). Indeed, mitochondria were found to be positioned at the cytosolic edge facing the ER, and in actual contact with ER Ca^2+^ transporters [95]. The MCSs enable the exchange of signals involving a set of various proteins and contribute to mitochondrial Ca^2+^ signalling due to the plausible uptake of ER-released Ca^2+^ through the MCU, possibly via IP_3_R and MCU-associated proteins [98,99].

#### 2.3.1. Mitochondrial Ca^2+^ Uptake Machinery

In the IMM, the MCU channel functions as a heteromeric protein complex of ~450–800 kDa that includes the ion-conducting core MCU protein and several MCU-associated regulatory proteins, including MCU dominant negative beta subunit (MCUb), essential MCU regulator (EMRE), the MICU family (MICU1, MICU2, MICU3), and MCUR1. Together, they constitute the mitochondrial uptake machinery (MCUT-M) [100,101].

The MCU was identified by two leading studies in 2011 [102,103]. The MCU is encoded by the CCDC109A gene and is a highly conserved 40 kDa protein ubiquitously expressed in metazoans. The human MCU is composed of four domains: the N-terminal domain, the helical linker domain, the coiled-coil domain, and the transmembrane domain [104]. The MICU1-dependent opening and closing of MCU was also successfully studied. MICU1 regulates the conformation of MCU via their EF-hand domain, which is a chemical sensor of Ca^2+^. Recently, the structure of the entire 480 kDa supercomplex indicates the main molecular interactions between the four major components: MCU–EMRE–MICU1–MICU2 [105]. At low Ca^2+^ concentrations, the MICU1–MICU2 heterodimer blocks the MCU channel, thus Ca^2+^ cannot accumulate inside the matrix. When Ca^2+^ concentration is increased, the EF-hands of MICU1 and MICU2 sense the change in Ca^2+^ level in the intermembrane space. These MICU proteins undergo conformational changes that dissociate the MCU holocomplex, thus allowing Ca^2+^ to enter the mitochondrial matrix [106].

Genetic manipulations of MCU revealed its crucial role in the regulation of mitochondrial Ca^2+^ signalling. Subsequently, MCU deficiency and overexpression were studied by many independent research groups to determine its role in evolution and pathophysiology. For instance, Huang et al., demonstrated that the loss of MCU decreased ATP production, whereas MCU overexpression increased mtROS production and apoptotic rate in *Trypanosoma brucei* [107]. In adult zebrafish, the nonsense mutant of MCU possessed a cardiomyopathy-like phenotype with reduced cardiac chamber size.

Transcriptomic analysis of MCU mutant zebrafish showed the deregulated expression of genes that are involved in potassium transport activity, Ca^2+^ transport, cell junctions, the and electron transport chain [108]. Moreover, *MCU* knockout mice displayed a smaller body size, with no changes in overall body composition. Mitochondria isolated from *MCU* knockout mice were not able to uptake Ca^2+^; strikingly, no basal metabolic processes were altered in the absence of MCU [109].

MCU function can be regulated by several proteins. Thus, MCUb (39 kDa) encoded by *CCDC19,* a gene expressed exclusively in vertebrates, plays the role of a negative regulator of MCU [110], the ratio between MCUb and MCU being crucial to maintain cell type specific Ca^2+^ homeostasis.

In addition, in the intermembrane space, MICU1 (54 kDa), encoded by *CBARA1*, establishes the threshold for MCU activity via its EF-hand Ca^2+^ binding domains [111]. MICU1 is the gatekeeper of mitochondrial Ca^2+^ uptake. This was demonstrated notably by the knockdown of MICU1 in HeLa cells, which have an increased Ca^2+^ accumulation, mtROS production, and apoptosis rate [112]. Moreover, in vivo knockout of *MICU1* in mice induced Ca^2+^ accumulation and altered mitochondrial morphology, resulting in decreased ATP production [113]. In mouse hepatocytes, MICU1 silencing failed to regenerate new liver cells, which was associated with an increase in mitochondrial permeability pore (mPTP) opening and massive necrosis [114]. Interestingly, MICU1 activity is regulated by mitochondrial pyruvate and fatty acid flux. Therefore, MICU1 plays a role as the metabolic checkpoint that protects cells from Ca^2+^ overload, thus preventing cells from bioenergetic crises and programmed cell death [113].

MICU2 is a 45 kDa protein that is ubiquitously expressed in mammalian cells. It is also located in the mitochondria intermembrane space, and physically interacts with MCU and MICU1 [115]. Sequence analysis revealed that the MICU1 gene shares approximately 25% sequence identity with the MICU2 gene and, similarly, MICU2 possesses two EF-hands acting as Ca^2+^ binding sites. MICU2 also acts as a gatekeeper, keeping the channel closed at low cytosolic Ca^2+^ levels [116]. MICU2 also restricts the Ca^2+^ crosstalk between IP_3_R in the ER and MCU in the mitochondria [117].

Another direct activator of MCU is *SMDT1*-encoded 10 kDa protein EMRE [118]. Without EMRE, the MCU channel was found in a monomer form, suggesting that EMRE has an important role in MCU dimerization and complex assembly [119]. Phenotypes of knockout *EMRE* are like that of knockout *MCU* in human embryonic kidney (HEK)-293T cells [118]. Neutralization of the binding site of MCU on EMRE led to EMRE binding to MICU1, and the inhibition of the MICU1 gatekeeping function [120].

Finally, the *CCDC90A* gene encodes a 35 kDa MCUR1 protein required for MCU-mediated mitochondrial Ca^2+^ uptake as a scaffold factor for MCU channel function. Its role in the assembly of the MCU supercomplex is also important [121]. The exact function of MCUR1 is still debated. Chaudhuri et al. hypothesized that MCUR1 mediates the opening of mPTP by controlling the Ca^2+^ threshold [122], whereas Paupe et al. demonstrated that MCUR1 directly regulates the cytochrome c oxidase assembly factor [123]. The loss of MCUR1 induces the incorrect assembly of the MCU complex, followed by a decrease in mitochondrial membrane potential (ΔΨ_m_).

Altogether, this emphasizes the importance of the correct assembly and coordination of the Ca^2+^ transportome to guarantee proper mitochondrial function.

#### 2.3.2. Other Transporters in Mitochondria

The voltage-dependent anion channel (VDAC) located at the OMM is responsible for the flux of several metabolites and small cations into the mitochondria, serving as the mitochondrial gateway, and ensuring the crosstalk between mitochondria and other cellular compartments [124]. OMM is permeable to Ca^2+^ via the VDAC, and Ca^2+^ itself plays a role as a negative feedback regulator of the VDAC [125]. The open state of the VDAC allows the flux of anions, whereas, in the closed state, the VDAC promotes the non-selective transport of cations, including Ca^2+^ [126]. The VDAC vitally contributes to cancer metabolism and metabolic reprogramming by determining the cytosolic ATP/ADP ratio, which either enhances or reduces the so-called Warburg effect. The VDAC is involved in almost all of the important metabolic processes, including the anabolism of amino acids, fatty acids, cholesterol, and glucose, thus promoting tumorigenesis [127]. Among its three isoforms (VDAC1, 2, 3), high expression of VDAC1 is associated with a negative outcome in terms of HCC [128]. VDAC1 directly interacts with the antiapoptotic Bcl2, hexokinase I (HK-I), and HK-II to protect cells against programmed cell death [129].

The solute carrier protein family 25 (SLC25) members are located at the IMM, and are responsible for transporting metabolites, nucleotides, and cofactors across the non-permeable IMM. SLC25 proteins contain EF-hands at the N-terminal domain, and are Ca^2+^-dependent; furthermore, they are involved in several metabolic processes and are identified as potential biomarkers of various cancers [130]. For example, the ATP-Mg^2+^ solute carrier SLC25A23 is an adenine nucleotide transporter that interacts with the MCU and MICU1 and enhances mitochondrial Ca^2+^ uptake. SLC25A23 knockdown reduces mitochondrial Ca^2+^ uptake rate and protects cells from oxidative stress due to mtROS production, as demonstrated in a HeLa cell line model [131].

Moreover, the mitochondrial Na^+^/Ca^2+^/Li^+^ exchanger (NCLX) is responsible for Ca^2+^ extrusion from the mitochondrial matrix into the intermembrane space (IMS). Each exported Ca^2+^ ion is exchanged with three imported Na^+^ [132]. The balance between the MCU (Ca^2+^ uptake) and the NCLX (Ca^2+^ extrusion) regulates Ca^2+^ homeostasis, thus impacting cellular metabolism and Ca^2+^-related signalling pathways. By extruding Ca^2+^ from the mitochondria, NCLX can impact OXPHOS and mtROS production [133]. Similar to other members of the mitochondrial Ca^2+^ transportome, NCLX can also contribute to many metabolic processes, such as ATP, fatty acid, and nucleotide synthesis, etc., [134]. Although the role of NCLX in cancer is less explored than other members of the mitochondrial Ca^2+^ transportome, it has been shown that genetic loss of *NCLX* in colorectal cell lines (HCT116 and DLD1) could cause mitochondrial Ca^2+^ overload and inhibits proliferation and increases migration and chemoresistance by transcriptional reprogramming. Accordingly, low NCLX mRNA levels are correlated with advanced tumour stages in colorectal cancer [134].

## 3. Deregulation of the Ca^2+^ Transportome and Consequences on HCC

In various cancer cell types, Ca^2+^ fluxes regulate tumour proliferation, invasion, metastasis, programmed cell death resistance, etc., [135]. Moreover, Ca^2+^ behaves as a signalling messenger between the mitochondria, ER, cytosol, and the nucleus, participating in tumour metabolic adaptation. Particularly in HCC, many Ca^2+^ transporters and exchangers are modulated, and can be classified according to their localization and expression level (Table 1).

Here, we specifically highlight the deregulation of some Ca^2+^ channels from three distinct cellular localizations: mitochondria, ER, and PM, to discuss their role in tumour progression in HCC patients and preclinical models.

### 3.1. Mitochondria Ca^2+^ Uptake Machinery (MCUT-M)

#### 3.1.1. Mitochondrial Ca^2+^ Uniporter (MCU)

In a pioneering study by Ren and colleagues, MCU mRNA and protein levels were found to be upregulated and correlated with lower overall survival and relapse-free survival, in 20 pairs of HCC patient tissues, compared with non-HCC tissues [145].

To elucidate the molecular role of the MCU and the consequences of its deregulation on HCC, the hepatocarcinoma cell lines SMMC-7721 and MHCC97H were used as models [145]. These models showed that the modulation of MCU protein expression levels impacts the NAD^+^/sirtuin-3 (SIRT3)/superoxide dismutase 2 (SOD2) pathway. *MCU* overexpression in MHCC97H cells increased the mitochondrial uptake Ca^2+^ under histamine stimulation, and significantly increased the enzymatic activities of several TCA enzymes, such as PDH pyruvate dehydrogenase (PDH), alpha ketoglutarate dehydrogenase (α-KGDH), and isocitrate dehydrogenase (IDH), and decreased the NAD^+^/NADH ratio. In contrast, the MCU downregulated SMMC-7721 cells showed opposite effects [145]. This influenced the levels of acetyl-CoA, α-ketoglutarate, and other cofactors, which are essential for the epigenetic enzyme functions that regulate transcriptional profiles and the epigenetic landscape. Therefore, this finding showed a link between metabolism and epigenetics in HCC [148,149].

In addition, the mitogen-activated protein kinase (MAPK) pathway appeared to be crucial for HCC cell migration and invasion by activating cJun NH2-terminal kinase (JNK), p38, and/or extracellular signal-regulated kinase (ERK) [150]. *MCU* overexpression in MHCC97H cells greatly increases phosphorylated JNK (p-JNK) levels but has no impact on the levels of p38 or ERK. Moreover, JNK downstream actors, such as p-Paxillin and matrix metalloproteinase 2 (MMP2), are also upregulated in *MCU*-overexpressed MHCC97H cells. In contrast, MCU downregulated SMMC-7721 showed opposite effects. P-Paxillin is implicated in the lamellipodia formation and focal adhesion turnover that facilitate tumour migration [151], whereas MMP2 is responsible for HCC cell invasion [152]. Futhermore, the implication of MCU-mediated mitochondrial Ca^2+^ in HCC metastasis was confirmed in an orthotopical nude mice model. *MCU* overexpressed mice exhibited a higher metastatic capacity, with a decreased NAD^+^/NADH ratio and an increased p-JNK level, in vivo.

**Figure 2:**
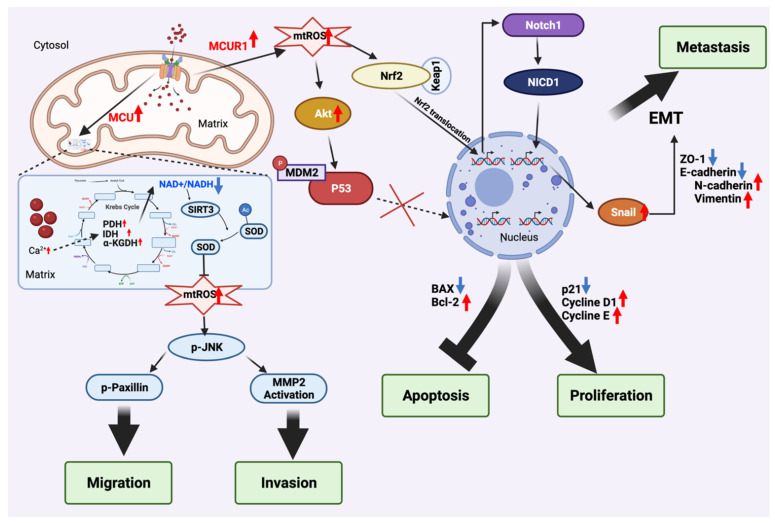
Deregulation of MCU and MCUR1-involved mitochondrial Ca^2+^ uptake in tumorigenesis and metastasis of hepatocellular carcinoma. Scheme of key actors involved in the retrograde signalling pathway from mitochondria to nucleus that promote HCC tumorigenesis. The increase in MCU-dependent mitochondrial Ca^2+^ results in the overproduction of mitochondrial ROS. This, in turn, inhibits NAD+/SIRT3/SOD2 pathway and stimulates the activation of ROS/JNK pathway that promotes HCC migration and invasion. Overexpression of *MCRU1* exhibits the same effect on ROS production but inactivates P53 via ROS/Akt/MDM2 pathway, promoting HCC proliferation and evading apoptosis (a cell death mechanism) by modifying transcriptional profiles of BAX, Bcl-2, p21, cyclin D1, and cyclin E. The activation of Snail-related EMT and ROS/Nrf2/Notch signalling pathway was also observed in *MCUR1* overexpressed HCC, thus inducing metastasis by transcriptionally upregulating N-cadherin and vimentin, and downregulating ZO-1 and E-cadherin. Figure created with BioRender.com (accessed on 20 January 2022).

Together, MCU-mediated mitochondrial Ca^2+^ excessively increases mtROS production via the NAD^+^/ SIRT3/ SOD2 pathway, thus promoting HCC invasion, as well as migration via the mtROS/JNK pathway (Figure 2).

#### 3.1.2. Mitochondrial Ca^2+^ Uniporter Regulator 1 (MCUR1)

Ren, Wang, and colleagues demonstrated, for the first time, that MCUR1 was overexpressed in 20 pairs of HCC patient tissues, compared with non-HCC tissues. Similar results were obtained from 128 HCC patient tissues by analysis using immunohistochemical staining [146]. MCUR1 mRNA and protein upregulation were associated with lower overall survival and relapse-free survival [146].

In line with HCC patient observations, the upregulation of MCUR1 facilitates HCC cell survival and tumour proliferation via ROS-dependent p53 degradation. *MCUR1*-overexpressed MHCC97H cell lines and xenograft tumours display higher growth capacity. In contrast, the accumulation of MCUR1 knockdown BEL7402 cells in G1 phase was also observed, indicating cell cycle perturbation and slower proliferation. As expected, this is associated with a high apoptotic rate both in cellulo and in xenograft model. [146].

Like MCU knockdown and overexpressed cell line models, the MCUR1 knockdown BEL-7402 cell line decreased the basal level of mitochondrial Ca^2+^ by inhibiting MCU-dependent Ca^2+^ uptake, whereas the *MCUR1* overexpressed MHCC97H cell line showed greater mitochondrial Ca^2+^ uptake capacity. Abnormal mtROS production was also observed in the *MCUR1* overexpressed MHCC97H cell line. mtROS overproduction may induce phosphorylation of Akt, thus stimulating the phosphorylation of MDM2. Phosphorylated MDM2 inhibits functional p53, whereas mRNA p53 level remains unchanged. Protein expression of p53 downstream targets, such as cyclin E, cyclin D1, and Bcl2, are increased, while p21 and Bax are decreased. This cascade promotes tumour proliferation by stimulating the cell cycle and avoiding apoptosis [146]. Thus, MCUR1 can indirectly inactivate P53 via ROS/Akt/MDM2 pathways and promote tumour proliferation in HCC models.

Recently, Jin and colleagues observed that MCUR1 was upregulated in metastatic HCC, by comparing 63 metastatic patient tissues with 74 non-metastatic patient tissues [147]. *MCUR1* upregulation promotes tumour proliferation and apoptosis avoidance, but also facilitates epithelial–mesenchymal transition (EMT) and metastatic capacity via the ROS/nuclear factor erythroid 2-related factor 2 (Nrf2)/Notch1 pathway in HCC, in both in vitro and in vivo models [147]. Moreover, *MCUR1* overexpressed MHCC97L cell lines displayed a significant elevation in mitochondrial Ca^2+^ and mtROS production, and facilitated the EMT, coinciding with a decreased expression of epithelial markers (E-cadherin and ZO-1) and an increased expression of mesenchymal markers (N-cadherin and vimentin), both in vitro and in an orthotopic transplantation model. MCUR1 downregulated BEL7402 displayed the complete opposite effects [147]. The epithelial and mesenchymal markers can be regulated by EMT transcriptional factors, such as Snail, which is crucial for the EMT-inducing pathway [153]. Indeed, *Snail* expression was significantly decreased by MCUR1 knockdown in HCC cell lines. A significant reduction in cytoplasmic Notch1 and nuclear NICD1 protein levels in the MCUR1 knockdown HCC cell line was also observed. The simultaneous inhibition of the Snail-related EMT and ROS/Nrf2/Notch pathway, via Nrf2 knockdown and using the Notch1 inhibitor DAPT, decreased MCUR1-induced EMT [147]. These results suggest the contribution of MCUR1-mediated mitochondrial Ca^2+^ in facilitating HCC EMT by overproducing mtROS, and thus mediates Nrf2 translocation and activates Snail-related EMT via Notch1 and its active form NICD1 [154] (Figure 2).

### 3.2. Plasma Membrane Channel—Transient Receptor Potential Cation Channel Subfamily C Member 6 (TRPC6)—Link with the TGFβ Pathway

Recently, eight HCC patient tissues were examined in the study of Xu and colleagues, showing that TRPC6 and NCX1 protein expression is significantly increased in HCC patient tissues compared to non-HCC tissues. Immunohistochemistry staining of 150 HCC patients confirmed that their expression levels were positively correlated with HCC malignancy degree [36].

In HCC cell lines (HepG2 and Huh7) and an HCC mouse model, transforming growth factor beta (TGFβ) stimulation (which elevates cytosolic Ca^2+^ levels via TRPC6 and NCX1 (28)) induces the intrahepatic metastasis of HCC; alternatively, knockdown of TRPC6 or NCX1 inhibits this metastatic development. Interestingly, after 24 h of TGFβ stimulation, EMT was observed with a decreased expression of epithelial marker E-cadherin and an increased expression of mesenchymal vimentin. Thus, both MCRU1 and the TRPC6/NCX1 complex are involved in the EMT mechanism [36]. Moreover, this TGFβ/TRPC6/NCX1 pathway did not change Snail mRNA expression, unlike the MCUR1-mediated EMT mechanism, suggesting an independent pathway that stimulates epithelial-to-mesenchymal transition of HCC.

When TGFβ binds to its receptor, TGFβ can phosphorylate and activate Smad2, which is known as a TGFβ downstream target and a transcription factor that participates in the regulation of several genes [155]. The stimulation of TGFβ during 2–4 h increases the phosphorylated form of Smad2 (pSmad2). Smad2 knockdown in Huh7 cells also reduces the expression of *TRPC6* and *NCX1*, suggesting that TGFβ induces the formation of the TRPC6/NCX1 complex via the phosphorylation of Smad2. On the other hand, knockdown of TRPC6 or NCX1 inhibited Smad2 phosphorylation, demonstrating a reciprocal positive feedback loop between Smad2 and the TRPC6/NCX1 complex in HCC cell lines [36].

Futhermore, several HCC multidrug resistance (MDR) mechanisms were demonstrated to be cytosolic Ca^2+^-dependent [156,157,158]. In a study conducted by Wen et al., Huh7 and HepG2 cell lines were exposed to doxorubicin, hypoxia, or ionizing radiation to generate HCC MDR models [35]. Using these models, these authors found that, under long-term stimulation, cytosolic Ca^2+^ was accumulated and *TRPC6* mRNA level was upregulated. Moreover, the BAPTA-AM intracellular Ca^2+^ chelator could inhibit the expression of all MDR-related mechanisms, suggesting that TRPC6 is responsible for Ca^2+^-dependent MDR in HCC [35]. TRPC6/Ca^2+^ signalling relates to MDR-related mechanisms via the transcription factor STAT3 [159]. Indeed, TRPC6 knockdown decreased p-STAT3 protein expression; in addition, the STAT3 inhibitor also decreased the MDR-related mechanism in MDR-induced Huh7 and HepG2 cell lines. Accordingly, shRNA-mediated TRPC6 inhibition increases tumour responsiveness to doxorubicin treatment, as shown by a five-fold tumour size reduction in the in vivo HCC xenograft compared to doxorubicin-only treatment [35].

**Figure 3:**
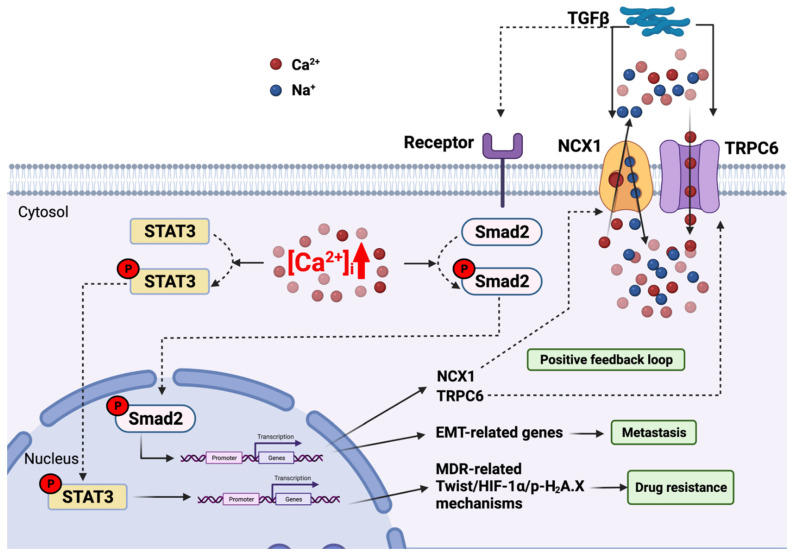
Deregulation of TRPC6-involved signalling pathways in hepatocellular carcinoma metastasis and drug resistance. Schema of key actors involved in the retrograde signalling pathway from mitochondria to nucleus that promote HCC metastasis and drug resistance. TGFβ can stimulate the activation of the TRPC6/NCX1 complex to increase cytosolic Ca^2+^ concentration. At the same time, TGFβ ligands activate their receptor complexes. Both Ca^2+^ concentration increase and TGFβ receptor activation stimulate the phosphorylation of Smad2, leading to the transcriptional upregulation of EMT-related genes that promote HCC metastasis, and of NCX1 and TRPC6 that create a positive feedback loop. The TRPC6-mediated high level of cytosolic Ca^2+^ can also induce the phosphorylation of STAT3, thus stimulating Twist/HIF-1α/H2A.X-related MDR mechanisms. Figure created with BioRender.com (accessed on 20 January 2022).

Taken together, these findings highlight the implication of TRPC6/NCX1-mediated cytosolic Ca^2+^ in TGFβ-driven EMT via the Smad2-dependent pathway, and in the MDR-related mechanism via the pSTAT3-dependent pathway. Further studies need to be conducted to verify the exact roles and molecular mechanisms of the TRPC6/NCX1 complex in different HCC stages, and TRPC6/Ca^2+^-dependent MDR mechanisms (Figure 3).

### 3.3. Endoplasmic Reticulum STIM1-a Metabolic Checkpoint Pathway in HCC

As introduced above, STIM1 is an ER Ca^2+^ sensor that mediates SOCE mechanisms, stimulating the influx of Ca^2+^ across the plasma membrane through Orai channels (Figure 1). Surprisingly, by analysing HCC patients and model data, recent studies revealed that STIM1 is upregulated in hypoxic HCC cells [160] and down regulated in metastatic HCC cells [142].

**Figure 4:**
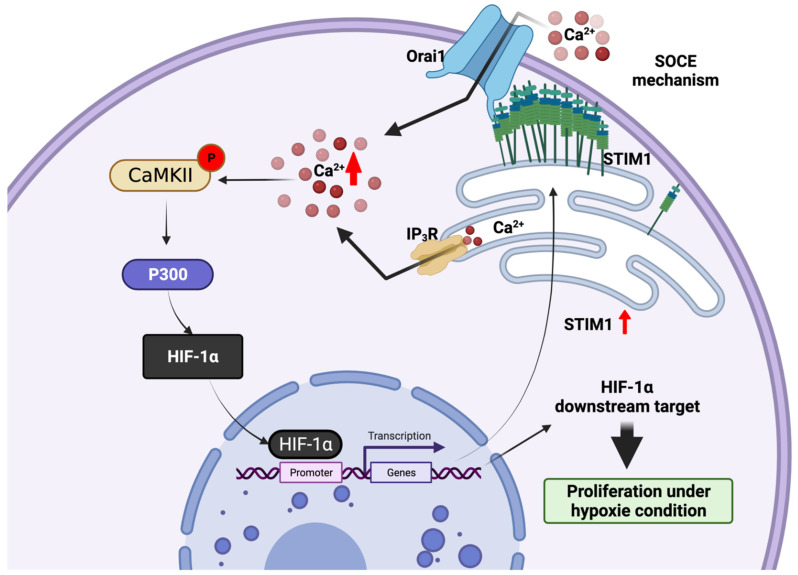
STIM1/PM SOCE, a metabolic checkpoint pathway in hepatocellular carcinoma. Schema of key actors involved in the retrograde/anterograde signalling pathway from ER to nucleus that promote metabolic HCC proliferation under hypoxic conditions. HIF-1α can bind to *STIM1* promoter and transcriptionally express STIM1. STIM1-mediated SOCE mechanism is activated by the interaction between STIM1 and Orai1. STIM1-mediated SOCE mechanism can also stabilize HIF-1α via SOCE/CaMKII/p300. The activated HIF-1α translocates into the nucleus and activates the transcription of its downstream target genes involved in HCC proliferation. Figure created with BioRender.com (accessed on 20 January 2022).

In hypoxic HCC cells, hypoxia-inducible factor-1 alpha (HIF-1α) is a transcription factor that plays important roles in hypoxic hepatocarcinogenesis. An analysis of 10 HCC patients revealed that the protein expression of STIM1 and HIF-1α was positively correlated in patient tissues and was also associated with the tumour size in a xenograft mouse model [151]. HIF-1α binds directly to the *STIM1* promoter and induces its transcription. Indeed, knockdown of HIF-1α in HepG2 cells reduced STIM1 expression and in vivo tumorigenesis, revealing the important implication of STIM1 in HCC proliferation. Interestingly, knockdown of STIM1 in HepG2 cells also decreased the HIF-1α level. STIM1-mediated cytosolic Ca^2+^ by the SOCE mechanism can stabilize HIF-1α by the activation of Ca^2+^/calmodulin-dependent protein kinase II (CaMKII) and p300 [160,161]. Together, a regulatory circuit consisting of the STIM1-mediated SOCE mechanism and phosphorylated p300-stablized HIF-1α can promote tumour proliferation under hypoxic conditions (Figure 4).

In metastatic HCC cells, immunohistochemical analysis of 12 HCC patients demonstrated that STIM1 protein level was downregulated and correlated with a lower overall survival of HCC patients [142]. In two *STIM1* knockout cell lines (SMMC7721 and HepG2), tumour invasion and proliferation were significantly inhibited. On the other hand, the resistance of anoikis that support HCC cell survival during metastasis was significantly increased in this model, suggesting the participation of STIM1-mediated cytosolic Ca^2+^ in anoikis [142].

Metastasis requires the metabolic switch from anabolism (ATP production by glycolysis and lipogenesis) to catabolism (fatty acid oxidation) to protect tumour cells against starvation and anoikis [162]. To examine the role of STIM1 in the metabolic reprogramming of metastasis, *STIM1* was knocked out in SMMC7721 and HepG2 cells. Remarkably, downregulation of glycolysis-involved genes (glucose transporter, 2/3-*GLUT2*/3; *HK2/3*; lactate dehydrogenase, A-*LDHA*; pyruvate dehydrogenase kinase, 1-*PDK1)* and de novo lipogenesis-involved genes (acetyl-CoA carboxylase, 1-*ACC1*; fatty acid synthase, *FASN*; ATP citrate lyase, *ACLY*) were observed. In contrast, fatty acid oxidation-involved genes (carnitine palmitoyl-transferase, A-*CPT1A*; long-chain acyl-CoA dehydrogenase, *LCAD)* were upregulated [142]. This finding suggests a metabolic checkpoint role of STIM1 that orchestrates HCC metastasis and anoikis resistance by metabolically switching from anabolism to catabolism.

Taken together, under hypoxic conditions, STIM1 is upregulated and mediates the SOCE mechanism. STIM1-mediated cytosolic Ca^2+^ entry by the SOCE mechanism stabilizes HIF-1α via CaMKII-mediated p300 activation, thus promoting tumour proliferation. In contrast, STIM1 protein level is downregulated in metastatic HCC cells. This leads to a shift of the metabolic balance from anabolism to catabolism, and contributes to metastasis and anoikis resitance.

## 4. Conclusions and Future Perspectives

This review describes some key roles of the Ca^2+^ transportome and its components in cancer, with an emphasis on HCC. Based on its various cellular locations (PM, ER, mitochondria), it is indisputable that the Ca^2+^ transportome influences interorganelle communication and can play a major role in cancer cell proliferation, metastasis, and drug resistance.

The understanding of Ca^2+^ transportome deregulation is increasingly expanding with regards to HCC initiation as well as progression [104]. Indeed, the overexpression or downregulation of members of the Ca^2+^ transportome have been identified in almost all stages of HCC development. Thus, in the future, deeper study of the MCU, MCUR1, STIM1, and TRPC6 in hepatocarcinogenesis may provide important information for the development of diagnostics and therapeutic approaches required to fight this aggressive and frequently relapsing cancer.

Finally, it can be speculated that future works will identify new components of Ca^2+^, and lead to a better understanding of the oncogenic deregulation of Ca^2+^-mediated interorganelle communication in HCC.

## Figures and Tables

**Table 1:** Dysregulated Ca^2+^ transportome in human HCC as potential targets.

Localization	Protein	Expression in HCC	References
Plasma membrane	CACNA1H	Upregulated	[136]
	TRPM2	Upregulated	[137]
	TRPV2	Upregulated	[138]
	TRPV4	Downregulated	[137]
	TRPC6	Upregulated	[35,36]
	TRPM7	Upregulated	[139]
	Orai1	Upregulated	[140]
Endoplasmic Reticulum	STIM1	Upregulated	[141,142]
	SERCA2	Downregulated	[143]
	SERCA3	Downregulated	[144]
	IP_3_R	Upregulated	[83]
Mitochondria	MICU1	Downregulated	[145]
	MCU	Upregulated	[145]
	MCUR1	Upregulated	[146,147]
